# *In silico* approach to understand epigenetics of POTEE in ovarian cancer

**DOI:** 10.1515/jib-2021-0028

**Published:** 2021-11-18

**Authors:** Sahar Qazi, Khalid Raza

**Affiliations:** Department of Computer Science, Jamia Millia Islamia, New Delhi 110025, India

**Keywords:** deep neural network, MD simulation, network-based epigenetics, ovarian cancer, POTEE, template-based modeling

## Abstract

Ovarian cancer is the third leading cause of cancer-related deaths in India. Epigenetics mechanisms seemingly plays an important role in ovarian cancer. This paper highlights the crucial epigenetic changes that occur in POTEE that get hypomethylated in ovarian cancer. We utilized the POTEE paralog mRNA sequence to identify major motifs and also performed its enrichment analysis. We identified 6 motifs of varying lengths, out of which only three motifs, including CTTCCAGCAGATGTGGATCA, GGAACTGCC, and CGCCACATGCAGGC were most likely to be present in the nucleotide sequence of POTEE. By enrichment and occurrences identification analyses, we rectified the best match motif as CTTCCAGCAGATGT. Since there is no experimentally verified structure of POTEE paralog, thus, we predicted the POTEE structure using an automated workflow for template-based modeling using the power of a deep neural network. Additionally, to validate our predicted model we used AlphaFold predicted POTEE structure and observed that the residual stretch starting from 237-958 had a very high confidence per residue. Furthermore, POTEE predicted model stability was evaluated using replica exchange molecular dynamic simulation for 50 ns. Our network-based epigenetic analysis discerns only 10 highly significant, direct, and physical associators of POTEE. Our finding aims to provide new insights about the POTEE paralog.

## 1 Introduction

Ovarian cancer is a slow and a silent killer in females leading to deaths annually [1–3]. Ovarian cancer that forms from the epithelial cells of the oviduct (fallopian tube) is very common in females. There are five types of ovarian cancer –growing from the epithelial cells (high grade/low grade serous), oviduct, endometrioid (endometrium), mucinous (cervical glands), and clear cell tumors (vaginal rests) [3, 4]. The World Health Organization (WHO) reports that ovarian cancer is detected in females in their 60s [5]. The disease is still an apex challenge for clinicians as its initial screening and diagnosis are not specific. There is a lack of effective biomarkers, and thus, no person-centric treatment strategy is available. Generic information suggests that age, familial history, genetics, environmental factors are responsible for causing ovarian cancer [2].

Epigenetics is a commonly encountered term with cancers and many other diseases and is simply a new sub-field in molecular biochemistry that aims to study the changed heritable physical characteristics (phenotypes), gene expression, and its activity without any change in the original DNA template sequence [6–8]. DNA methylation and histone modifications are the two widely studied epigenetic mechanism [9–13]. The epigenetic modifications consist of mutualistic interactions between DNA methylation, histone modification, and micro RNA (miRNA) expression that channelize and maintain the gene expression during cancer formation [14]. Myriad epigenetic mechanisms have also been observed to trigger the development of ovarian cancer [2]. Prostate ovary testis embryo expression (POTE), a cancer testis antigen (CTA) family of 14 paralogs have been classified according to phylogenetic evidences into three main groups – group I (POTEA), group II (POTEB1, B2, B3, C & D) and group III (POTE, F, I, J, KP, M) [15–18].

Researchers [17] in their study state that POTE groups I & II showed their normal testis-specific behavior in normal tissues where they expressed as cancer–testis antigens (CTAs) howbeit, POTE group 3 was observed in many normal tissues pointing to their non-CTA nature. In another study [19], the research group discerned that cancer testis antigen (CTA) family – POTE (prostate ovary testis embryo expression) has been intertwined to display its role in ovarian cancer due to global hypomethylation of L1 and 5′ CpG hypomethylation. They suggested that POTEs C, E, and F have a high dominance in high-grade serous epithelial ovarian cancer (HGSOC) combined with hypomethylation at 5′ promoter regions that they deduced from patient matched samples. While examining decitabine treatment and DNA methyl transferase (DNMT) knockout cell lines they validated that DNA methylation functions as a suppressor to POTE expression, while epigenetic drug treatment aiming histone deacetylases (HDACs) and histone methyltransferases (HMTs) along with decitabine improved the POTE expression. Also, Wang et al. [20] have screened POTEE paralog, viz., a group 3 member of the POTE family, and suggest its clinical importance to be used as an identifier for non-small cell lung cancer (NSCLC). With all these studies in hand, we aim to identify the lesser-known POTEE paralog in ovarian cancer using an exploratory *in silico* pipeline. With the few literature sources cited above that showcase that POTEE gets hypomethylated (over-expressed) in ovarian cancer, our study has validated this using an *in-silico* analysis that uses genomic, structural, electrostatic and epigenetics-based network approach. Genomic analyses help us to hint out at the correlation between the CTCF based motifs and POTEE paralog. We also predict the structure of POTEE using a deep neural network (DNN) based homology modelling and then compare it with existing Swiss-Model and AlphaFold POTEE models to check for the confidence per residue. We also check for the energy stability and electrostatic stability of our predicted model using replica exchange molecular dynamics (REMD). Finally, to establish why POTEE paralog showcases an epigenetic nature in ovarian cancer, we adopt a network based epigenetic approach that lists out the highly significant, direct and physical associators of POTEE.

## 2 Materials and modus operandi

### 2.1 Sequence retrieval

For analyzing the POTE ankyrin domain family member E(POTEE) we utilized the nucleotide sequence (mRNA) (Accession ID: NM_001083538.3) available on NCBI (https://www.ncbi.nlm.nih.gov/), while, for proteomic and network analyses we deployed the protein sequence (UniProt ID: Q6S8J3) available on UniProt (https://www.uniprot.org/).

### 2.2 Genomic analysis – motif identification, enrichment, comparison & occurrences

We used CTCFBSDB software [21, 22] for motif identification. Please see, we have not incorporated any statistical analyses in the current study. For CTCFBSs, we deployed a web-based tool named – CTCFDB that predicts the CTCF using different permutations and combinations of zinc fingers to identify divergent DNA sequences. This web tool has an array of identified core motifs for CTCFBS sequences and the motifs are shown using position weight matrices (PWM). In total, six PWM are used to represent CTCFBS sequences that get rectified and ultimately get included in the webtool repository. The EMBL_M1 and EMBL_M2 motifs were identified by Schmidt et al. [23], while the Ren_20 motif was first given by Kim et al. [24]; and the LM2, LM7, and LM23 motifs were rectified first by Xie et al. [25]. This webtool uses the STORM program15 and each of the six PWM to provide the best single sequence in the users query sequence. MEME suite’s CentriMo software [26] was deployed for motif enrichment purposes. TomTom software viz., available in MEME suite [27] was used for identified and enriched motif comparison. Find Individual Motif Occurrence (FIMO) was also deployed to identify the motif occurrences in the POTEE mRNA sequence [28]. We have used the threshold scoring parameter for selecting the best CTCF-based motifs, while *q* and *p*-values for selecting motif occurrences.

### 2.3 Structure prediction using a deep neural network approach

We used the POTEE protein sequence (UniProt Id: Q6S8J3) to a structure prediction analysis using the TopSuite web server [29]. For prediction purposes, only 5 main ankyrin repeats – ANK1 (172-201), ANK2 (205-234), ANK3 (238-267), ANK4 (271-300), and ANK5 (304-333) along with the main actin-like region that starts from residue 702-1075. The major loopy region (a) Loop 1 (399-435, length – 37 aa) and (b) Loop 2 (642-698, length – 57) making a count of 94 residues were ignored. The suite encapsulates the TopModel tool that predicts the protein structure using a top-down consensus approach to help the template selection. Also, it deploys the TopScore tool to evaluate the predicted models obtained.

### 2.4 Structural alignment: DNN-based POTEE model aligned to Swiss-Model POTEE model

We aligned our predicted POTEE model to the existing Swiss-Model [30] POTEE model (ID: Q6S8J3) to infer the common regions protein sequence (UniProt Id: Q6S8J3). This alignment of both the structures was executed in PyMol software [31]. Similarity index (%age), coverage and TM score were basic parameters that were used for selecting template that was to be used to develop the structure of POTEE.

### 2.5 Replica exchange molecular dynamic simulation (REMD) and electrostatic analyses

The modeled POTEE structure was submitted for the replica exchange molecular dynamics (REMD) in NAMD-VMD software [32] for 50 ns. CHARMM 22 parameter forcefield (par_all22_prot_cmap.inp) was deployed to compute the essential forces and energies for this purpose (https://www.ks.uiuc.edu/Training/Tutorials/namd/namd-tutorial-unix-html/node25.html). The maximum and minimum temperature ranges were obtained from temperature predictor for parallel tempering simulations viz., a webserver that generates temperature sets for REMD simulations [33]. The retrieved temperature string was as – 300.00, 318.87, 338.60, 359.16, 380.76, 400.00 (300–400 K) for 20 replicas. The model was minimized using the conjugate gradient (CG) algorithm [34]. The replica exchange desired acceptance ratio was tuned to be greater than 0.2 with the neighboring replica exchanges were checked after every 10 ps. A total of 20,000 replica exchanges were obtained after the completion of the simulation. 0.002 ps was set as the integration step for mass production run. The simulation time was set as 50 ns wherein the early 10 ns was kept for the equilibration phase and the remaining 40 ns for all of the additional analyses. Solvation was executed using a dodecahedron rhombic box where the shortest distance between the POTEE model and the edge of the box was kept 1 nm, thus 50,000 interacting particles in the entire system. Neutralization was done at 0.15 M NaCl concentration to maintain the overall charge of the system. Electrostatic associations were computed for each of the above mentioned steps using the particle-mesh Ewald (PME) method with a 1.2-nm cut-off range of electrostatic interaction. A cut-off of 1.2 nm was subjected to Lennard–Jones (LJ) interactions. Molecular mechanics generalized Born surface area (MM-GBSA) approach was used for calculating the binding free energy (delta G) over simulation time that was achieved by the adaptive Poisson–Boltzmann solver (APBS) plugin that is installed directly in PyMOL (https://pymolwiki.org/index.php/APBS_Electrostatics_Plugin). Moreover, Bluues software [35] was used for electrostatic calculations and surface potentials computations. The SCFbio ROG web tool [36] was deployed to calculate the radius of gyration (ROG). Protein Frustratometer 2 web server [37] was used to check and compute the energy landscape and dynamics. For REMD analysis, we relied upon the RMSD, accuracy scores, Molprobity, GBSE, total energy and ROG as the crucial parameters for assessment of the refined POTEE model.

### 2.6 Network analysis

We employed the protein sequence of POTEE paralog (UniProt ID: Q6S8J3) for the identification of protein interactors using ConsensusPathDB [38]. Network associators were selected based on the closest distance neighbour of POTEE paralog and thus was the main parameter in selecting and sorting the significant network associators.

## 3 Results

### 3.1 Genomic analysis

#### 3.1.1 Motif identification

To rectify different motifs present in the POTEE mRNA sequence we deployed CTCFBSDB software viz., based on CCCTC-binding factor (CTCF) that is simply a conserved transcription regulator ubiquitous in almost every organism – from the fruit fly to human beings. It attaches itself to various DNA sequences with the aid of 11-zinc fingers that mainly depend upon the biological context. These binding factors represent diverged DNA sequences and have been addressed to have a crucial part in gene expression control. Recent studies suggest that these factors are affiliated to genomic imprinting and X-chromosome inactivation [21, 22] that are two major epigenetic mechanisms. We submitted the mRNA sequence of the *Homo sapiens* POTEE (accession Id: NM_001083538.3) to CTCFBSDB and identified six essential motifs. Only three motifs namely (i) CTTCCAGCAGATGTGGATCA (score 13.1291), (ii) GGAACTGCC (score 12.1573), (iii) CGCCACATGCAGGC (score 8.44245) had the maximum likelihood to be present in the nucleotide sequence of POTEE as these hits were matching to various other motifs present in the repositories such as JASPAR 2020 [39]. Table 1 represents the identified motifs along with their confidence score in detail.

**Table 1:** Six important motifs identified in POTEE mRNA sequence.

S. No.	Motif sequence	Symbol	Start point	Length	Strand	Score
1	CGCCACATG CAGGC	M1	363	14	−	8.4424
2	GGAACTGCC	M2	3276	9	+	12.157
3	GGTGCCGCC AGACAGCAC TG	M3	3617	20	−	1.0849
4	CAGCCAGGA GAAGCCAGT A	M4	599	19	−	4.9250
5	CTTCCAGCA GATGTGGAT CA	M5	3780	20	+	13.129
6	CTTCCAGCA GATGTGGAT CA	M6	3780	20	+	7.9642

#### 3.1.2 Motif enrichment and occurrence analyses

**Figure 1:**
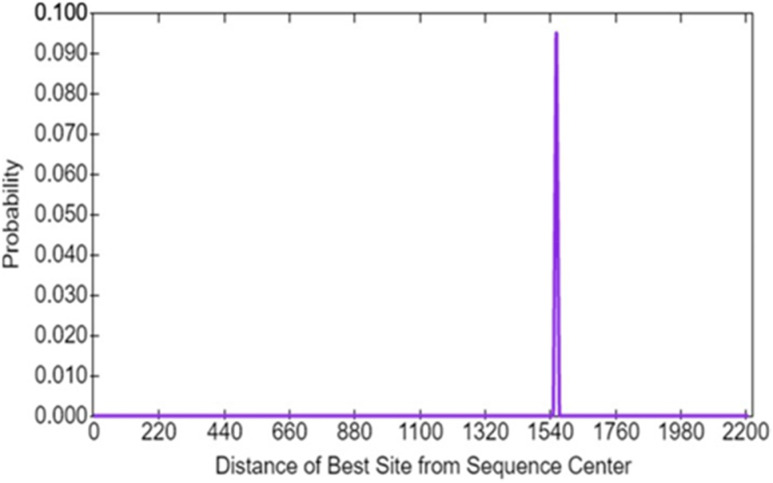
Motif probability graph showing the distance of the best site of the motifs from sequence center as retrieved by CentriMo software. 1540 is the location of the best-enriched motif with a significance score of 0.100.

The CentriMo predicted the nucleotide percentage present in the mRNA POTEE sequence. Nucleotide pair of A–T was 0.2534 while the C–G pair had 0.2466. Figure 1 is the predicted motif probability graph showing the distance from the best site from the sequencing center.

The three best scoring motifs were submitted for motif comparison using TomTom. For motif enrichment purposes, the Pearson correlation coefficient was used to score the motifs. All these motifs were perfect matches to other motifs in the human and mouse genome. For the M1 motif, 25 motifs were perfect matches, while for the M2 motif only 6 perfect matches were obtained. For the M5 motif, 13 matches were predicted using various databases namely, JASPAR2018_CORE_vertebrates_non-redundant, where 579 motifs were screened and only 23 were matched. In the uniprobe_mouse database, 386 motifs were screened that resulted in only 3 matches, whereas, for the jolma2013 database, 843 motifs were screened that resulted in 15 perfect matches to our query motifs – M1, M2, and M5. Depending on the E and P values, we have selected only the best scoring motif matches for all the three scoring motifs – M1, M2, and M5. These motifs were matches to zinc finger factors, DNA-binding domains, and transcription factors (TFs) present in both the human and the mouse genome. The enrichment analyses results have been given as a Supplementary Table S1. To find the motif occurrences in the mRNA sequence of POTEE, we used FIMO software. We set the parameter for *Homo sapiens*, selected the UCSC database (hg38). There were 64,488 motif occurrences with a *p*-value less than 0.0001. The best match motif was identified to be CTTCCAGCAGATGT which has a width of 14. Table 2 represents the top 20 motif occurrences that have been computed for the POTEE mRNA sequence.

**Table 2:** Occurrences of the motif throughout the human genome.

Gene name (chromosome)	Strand	Start-end	*p*-Value	*q*-Value	Matched sequence
APOA1 (chr11)	−	1205860-873	2.06e-09	0.192	CTTCCAGCAGATGC
ERRFI1 (chr1)	−	2939984-997	2.06e-09	0.192	CTTCCAGCAGATGC
OBSL1 (chr2)	−	2971741-754	2.06e-09	0.192	CTTCCAGCAGATGC
SPDL1 (chr5)	−	4124476-89	2.06e-09	0.192	CTTCCAGCAGATGC
ARHGEF25 (chr12)	–	5043900-913	2.06e-09	0.192	CTTCCAGCAGATGC
RBMY1A1 (chrY)	−	13640368-381	2.06e-09	0.192	CTTCCAGCAGATGC
ESRI (chr6)	+	15338772-785	2.06e-09	0.192	CTTCCAGCAGATGC
KIF14 (chr1)	−	19908856-869	2.06e-09	0.192	CTTCCAGCAGATGC
VIRMA (chr8)	+	20941333-346	2.06e-09	0.192	CTTCCAGCAGATGC
RC3H2 (chr9)	−	23097430-443	2.06e-09	0.192	CTTCCAGCAGATGC
BMI1 (chr10)	+	29578434-447	2.06e-09	0.192	CTTCCAGCAGATGC
MIR155 (chr21)	+	32639715-728	2.06e-09	0.192	CTTCCAGCAGATGC
SPDL1 (chr5)	+	32947709-722	2.06e-09	0.192	CTTCCAGCAGATGC
HMGB1 (chr13)	+	34897404-417	2.06e-09	0.192	CTTCCAGCAGATGC
SPHK1 (chr17)	−	42704182-195	2.06e-09	0.192	CTTCCAGCAGATGC
PMM1 (chr22)	−	47284180-193	2.06e-09	0.192	CTTCCAGCAGATGC
TFCP2 (chr12)	−	61516227-240	2.06e-09	0.192	CTTCCAGCAGATGC
RNF164 (chr9)	+	74024361-374	2.06e-09	0.192	CTTCCAGCAGATGC
YAP1 gene (chr11)	+	86977524-537	2.06e-09	0.192	CTTCCAGCAGATGC
ZNF684 (chr1)	+	88247890-903	2.06e-09	0.192	CTTCCAGCAGATGC

### 3.2 Deep neural network-based structure prediction

**Figure 2:**
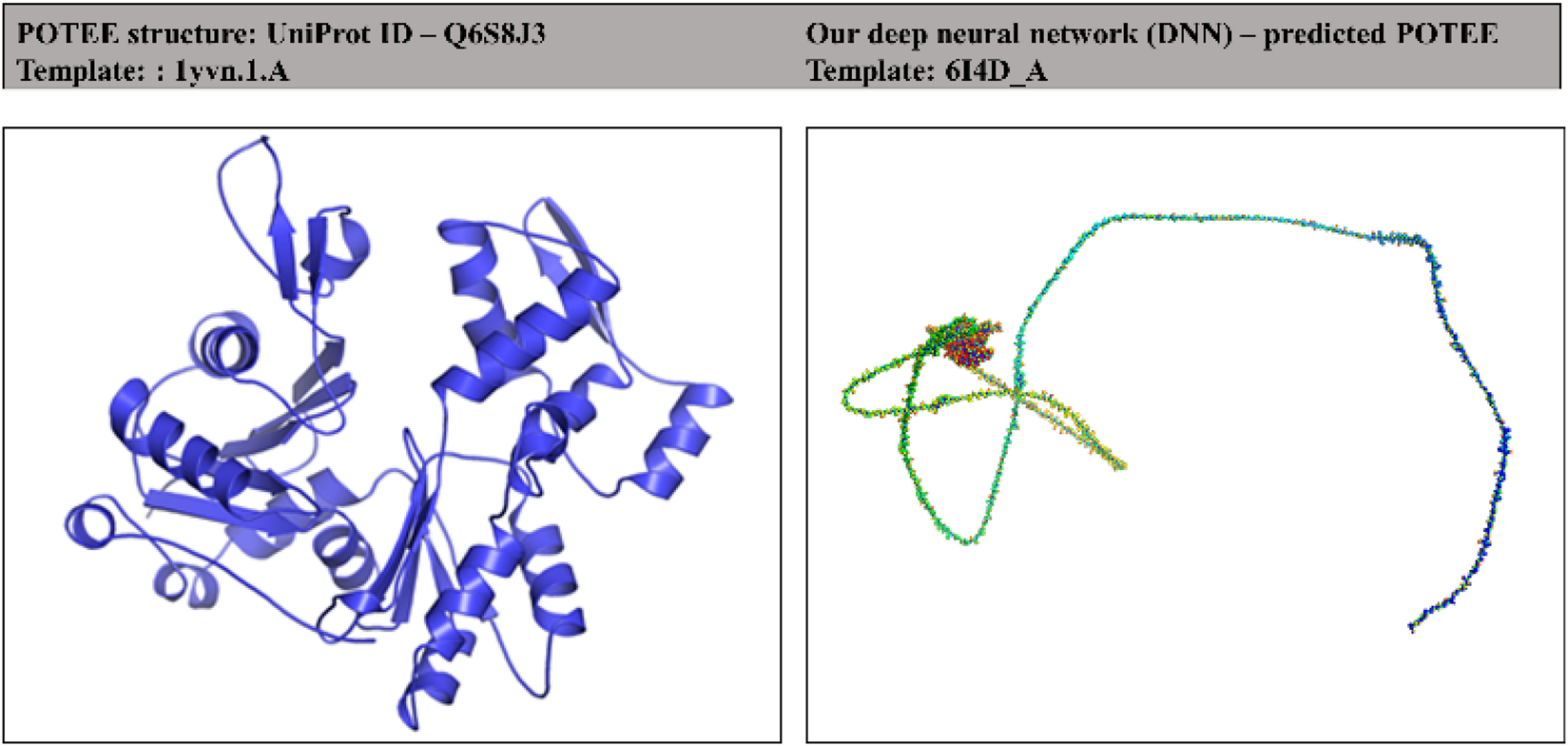
POTEE tertiary models. (A) An actin-like domain model of POTEE in Swiss-Model developed by template 1yvn.1. (A and B) Complete POTEE predicted model using DNN-based modeling software – the blue-colored regions represent a higher possible region while the red one’s correlate to low possible modeled regions.

The tertiary model of POTEE paralog has already been developed using the 1yvn.1. A PDB template and can be easily accessed and downloaded from Swiss-Model [30]. The model is a theoretical one with no experimentally validated crystal structure. The Swiss-Model POTEE structure showcases the actin-like domain and not the complete protein structure. We deployed the TopModel tool to predict the POTEE structure as it has an embedded automated workflow for template-based modeling (TBM) that uses the power of deep neural network (DNN) learning to improve template selection, thus, preparing the best possible and robust models with good overall quality, coverage and similarity index to the template models. Figure 2 represents the two varied structures of POTEE paralog – (a) a Swiss-Model structure (UniProt ID: Q6S8J3) and (b) our deep neural network predicted model using TopModel software. There is a magnanimous difference between the two models predicted. Our predicted model has been predicted based on the best matching PDB template 6I4D_A. In the predicted POTEE model, the blue-colored residues represent low predicted error referring to their high modeled quality, while red-colored residues correspond to high predicted error meaning they have a poor modeled quality.

**Figure 3:**
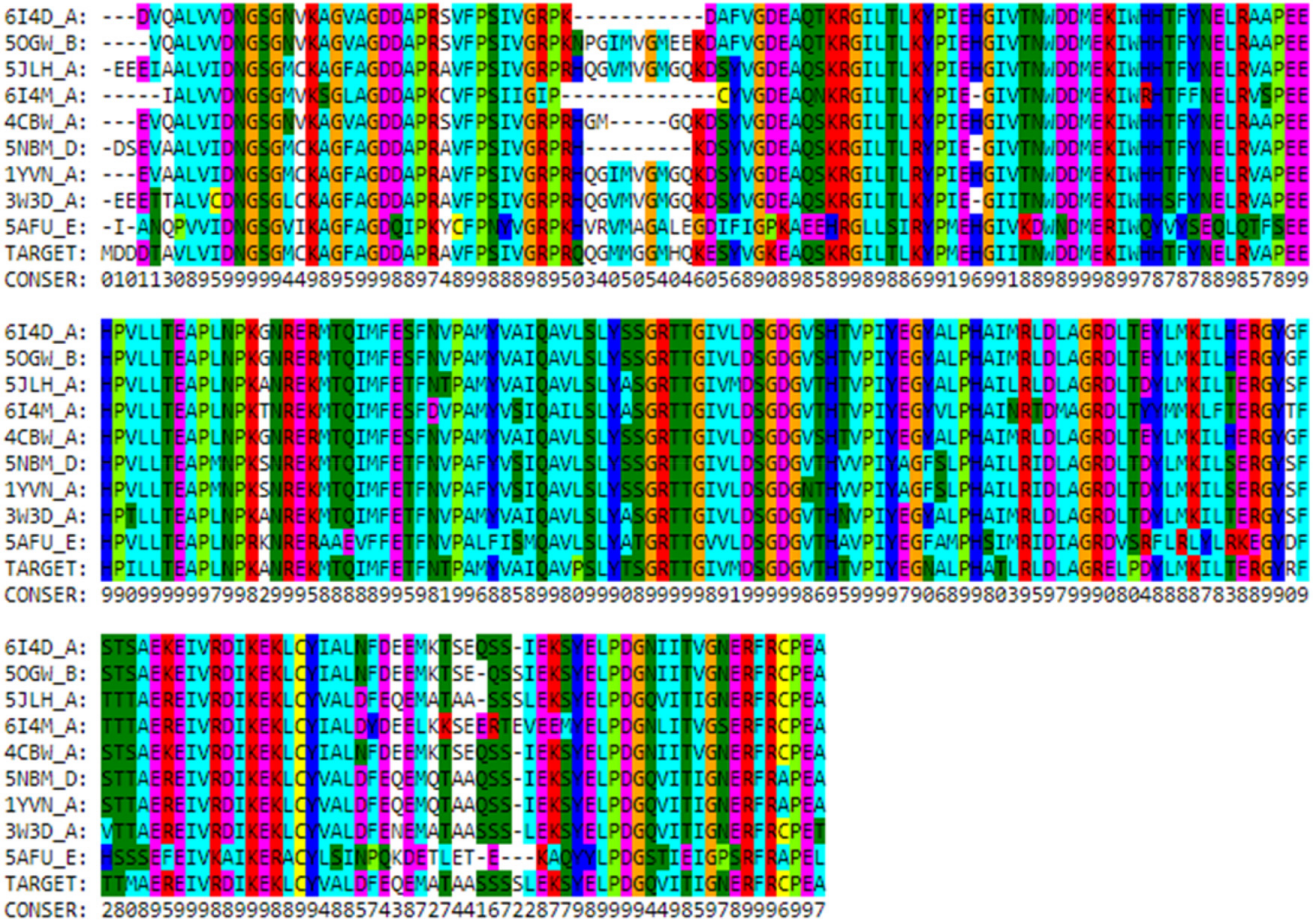
Multiple sequence alignment of the templates selected by DNN for model prediction.

Out of 50 templates, our deep neural network (DNN)-based approach selected only 5 best possible templates for POTEE tertiary structure prediction. Table 3 encapsulates the top 5 templates along with the coverage, similarity index, overall quality, and a TM score that is calculated by various neural networks that use information about the threading energy, structural similarity, and model quality predictions. Figure 3 showcases the multiple sequence alignment of the templates along with the number of conserved residues that were selected by deep neural network (DNN) for model prediction.

**Table 3:** Top 5 best matching templates selected for structure prediction.

Template (PDB Id)	Similarity index (%)	Coverage (%)	TM score (%)
*6I4D_A*	78.1	26.7	88.3
*5OGW_B*	78	26.7	88.2
*5JLH_A*	85.9	25.9	87.6
*6I4M_A*	74.3	25.1	87.6
*4CBW_A*	81.3	26.3	87.2

**Figure 4:**
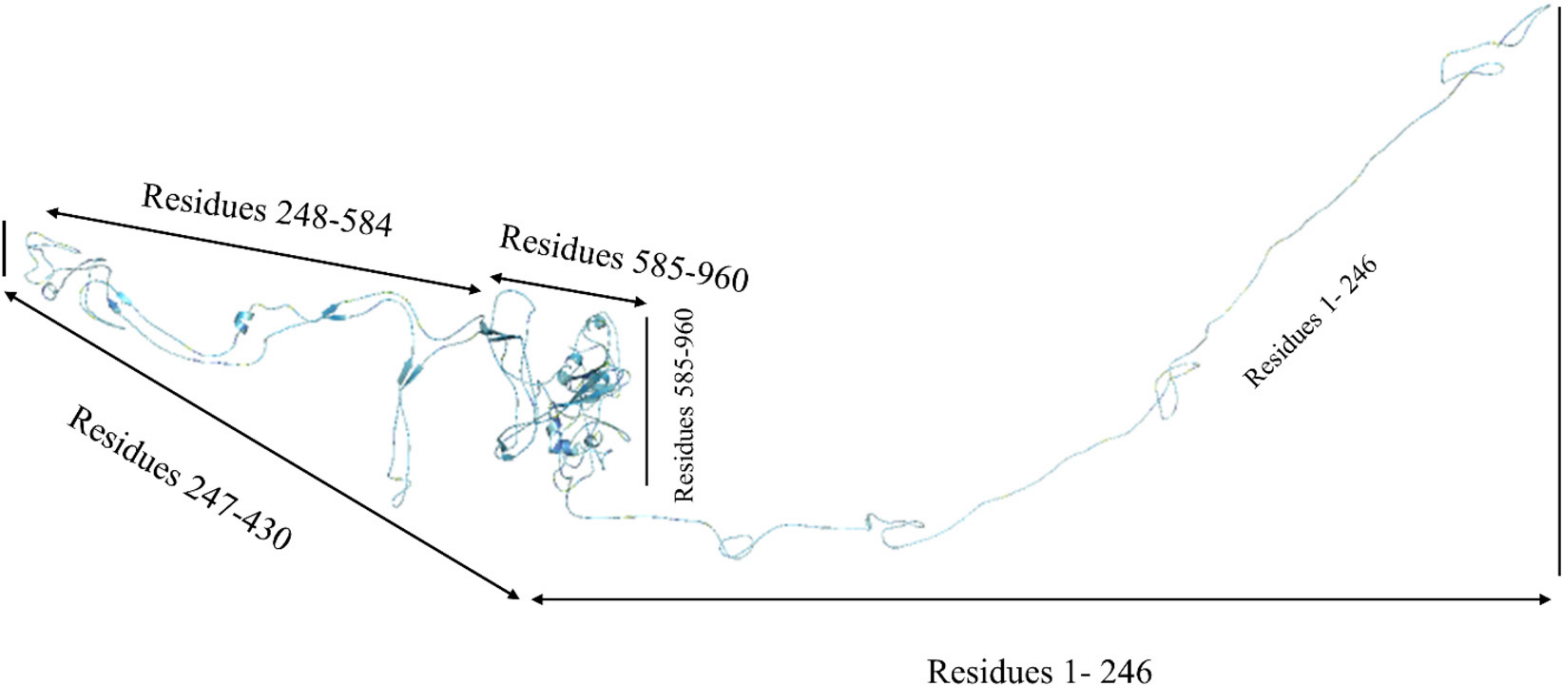
DNN-based predicted model of POTEE paralog.

The predicted model of POTEE has a single chain A with a length of 1–960 residues. Figure 4 represents the modeled structure of POTEE paralog with the residues that formed helices, parallel and anti-parallel strands, and loopy regions. With this, it is evident that our predicted model encapsulates the major domains and motifs of the POTEE paralog. Also, the main regions of functionality start from residue 248 and end at residue 960. Loops are formed from residue 1-246.

**Figure 5:**
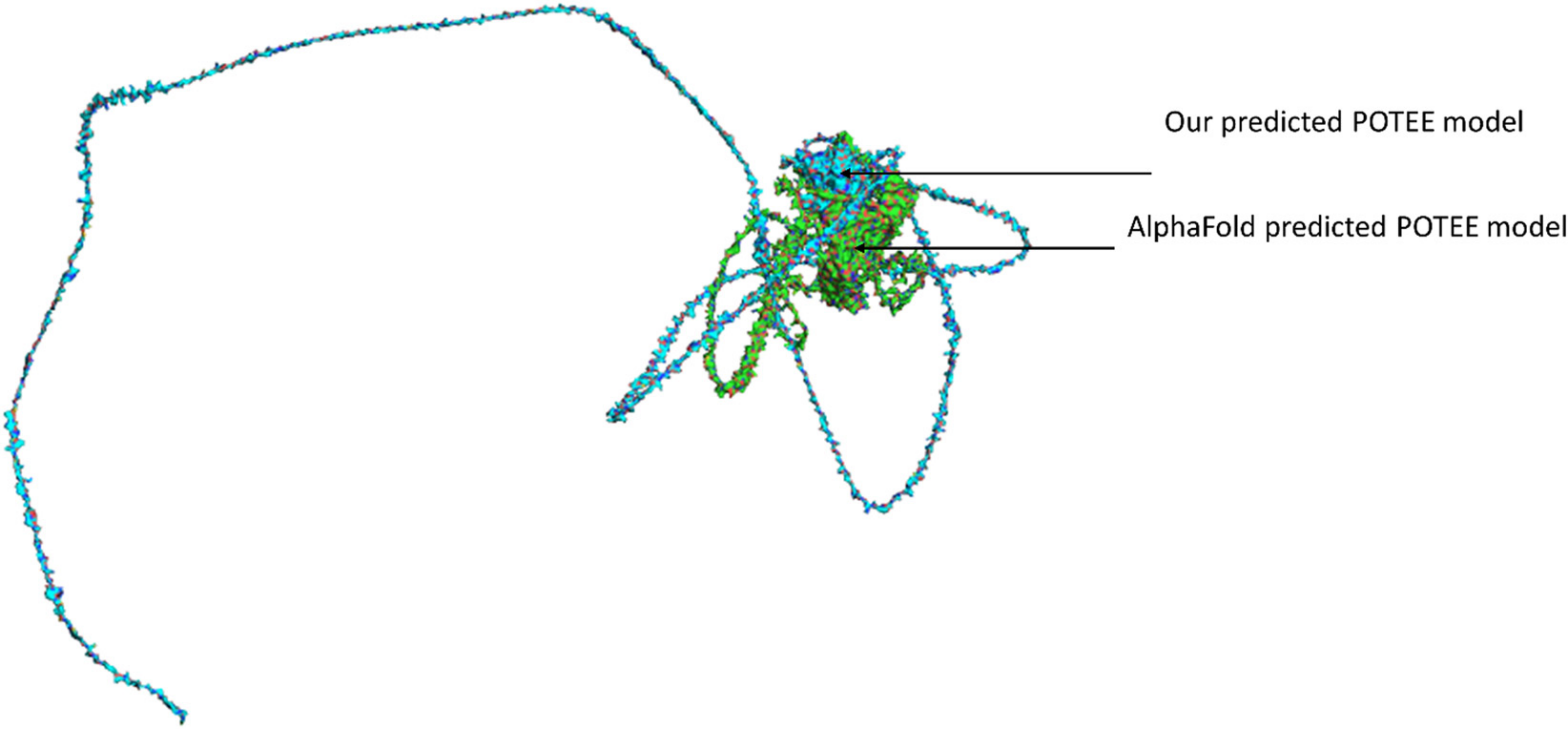
Validating predicted POTEE model with AlphaFold POTEE model.

To validate our predicted model, we utilised AlphaFold [40] structure predicted POTEE model (https://alphafold.ebi.ac.uk/entry/Q6S8J3). We downloaded the AlphaFold predicted model for POTEE that starts from residue 1 and ends are residue 1072. We aligned our predicted model to AlphaFold predicted model to check how well our predicted model has been developed (refer Figure 5). We observed that there was a perfect alignment to both the structures from residue 237-958, that corresponds to the fact that this segment of our predicted model has a very high confidence per residue i.e., >90. While, residues starting from 1-236 and stretch of residue starting from 959-1071 didn’t align well, that simply refers to having a poor per residue confidence score, i.e., <50.

### 3.3 Structural alignment

**Figure 6:**
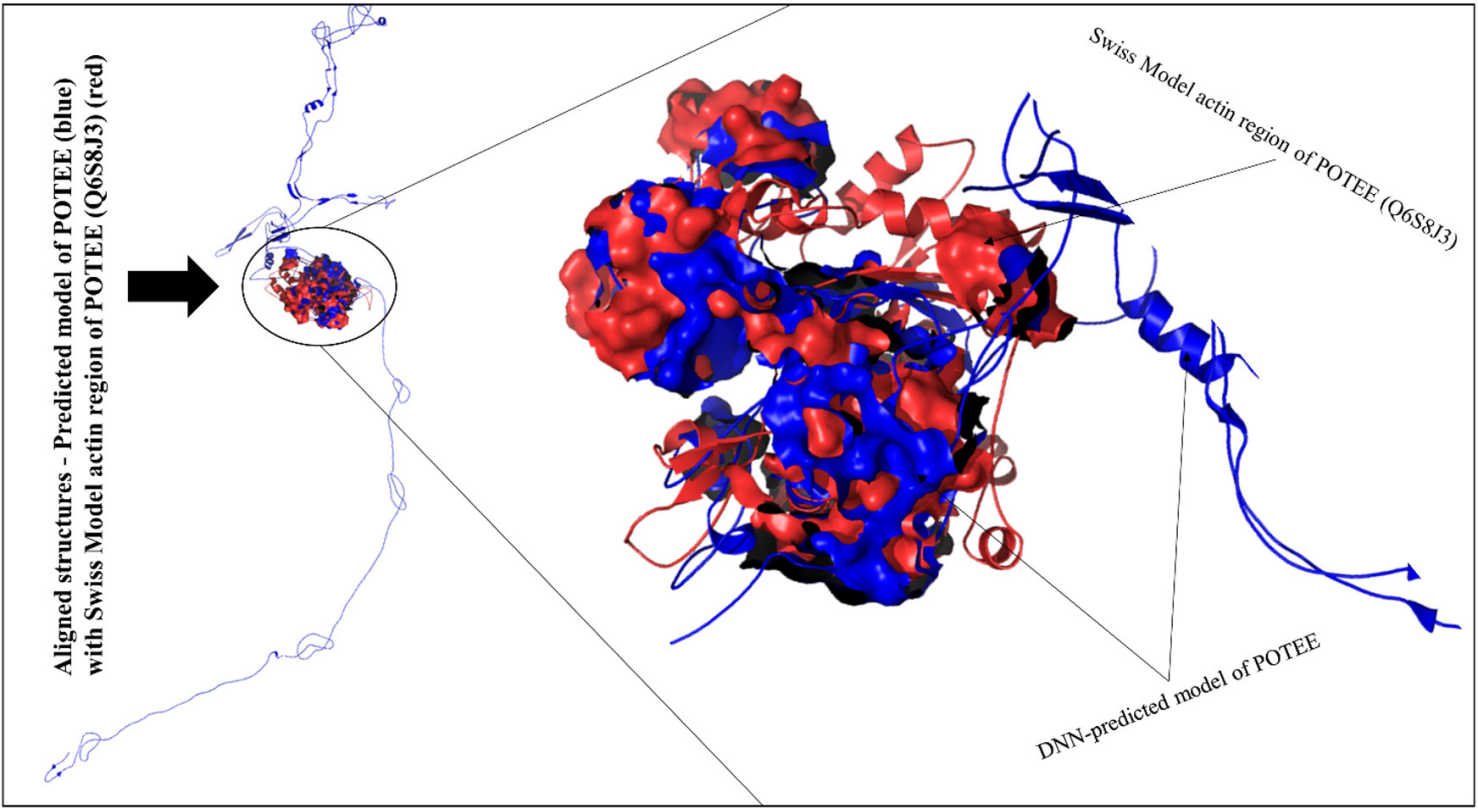
Aligned POTEE structures – DNN-predicted POTEE model (blue) aligned with Swiss-Model POTEE structure (red). The perfectly aligned regions have been represented in surface representation.

In order to see how it is different from the existing actin structure of POTEE paralog available in the Swiss-Model, we aligned both the structures – our deep neural network (DNN)-based predicted model of POTEE with the actin region of POTEE that is available in the Swiss-Model repository (Q6S8J3). Figure 6 represents the two aligned structures. It is evident that our predicted model of POTEE and the Swiss-Model actin POTEE region that starts from 705-1075 were aligned mainly at 5 intersections; residues 6-12 of predicted POTEE was perfectly aligned to 706-712. Resides 26-28, 66-76, and 81-140 in the predicted POTEE model were perfectly aligned to 726-728, 767-777, and 781-841 residues of the Swiss-Model POTEE structure. The longest aligned match portion started from residue 179-250 in our predicted model of POTEE with residue 879-949 of Swiss-Model POTEE structure.

### 3.4 Replica exchange molecular dynamic simulation (REMD) and electrostatic analyses

It is quite noticeable that the POTEE model has been refined with its overall energy being stable alongwith a good overall root mean square deviation (RMSD) score with minimum steric clashes. Table 4 provides the important parameters and temperature ranges for the REMD simulation run for our modeled POTEE paralog. Table 5 describes the temperature, energies and the probability exchange rate.

**Table 4:** Important factors for running REMD simulation.

Variable	Value
*P*_des_	0.2
Temperature range	300–400
Number of water molecules	0
Number of protein atoms	1075
Number of hydrogens in protein	∼552
Number of constraints	∼552
Number of virtual sites	∼1054
Number of degrees of freedom	∼1619
Energy loss due to constraints	6.68 (kJ/mol K)

**Table 5:** Different temperatures, energies and probability exchange rate required for replica exchange molecular dynamic simulation (REMD) of modeled POTEE.

S. No.	Temperature (K)	*μ* (kJ/mol)	*σ* (kJ/mol)	*μ*_12_ (kJ/mol)	*σ*_12_ (kJ/mol)	*P*_12_
1	300	−22,211	82.91	–	–	–
2	318.87	−22,064	85.17	147.2	118.86	0.2001
3	338.60	−21,910	87.53	153.9	122.13	0.2001
4	359.16	−21,750	89.99	161.0	125.54	0.1999
5	380.76	−21,581	92.58	168.3	129.11	0.2001
6	400.00	−21,431	94.88	150.2	132.56	0.2754

**Figure 7:**
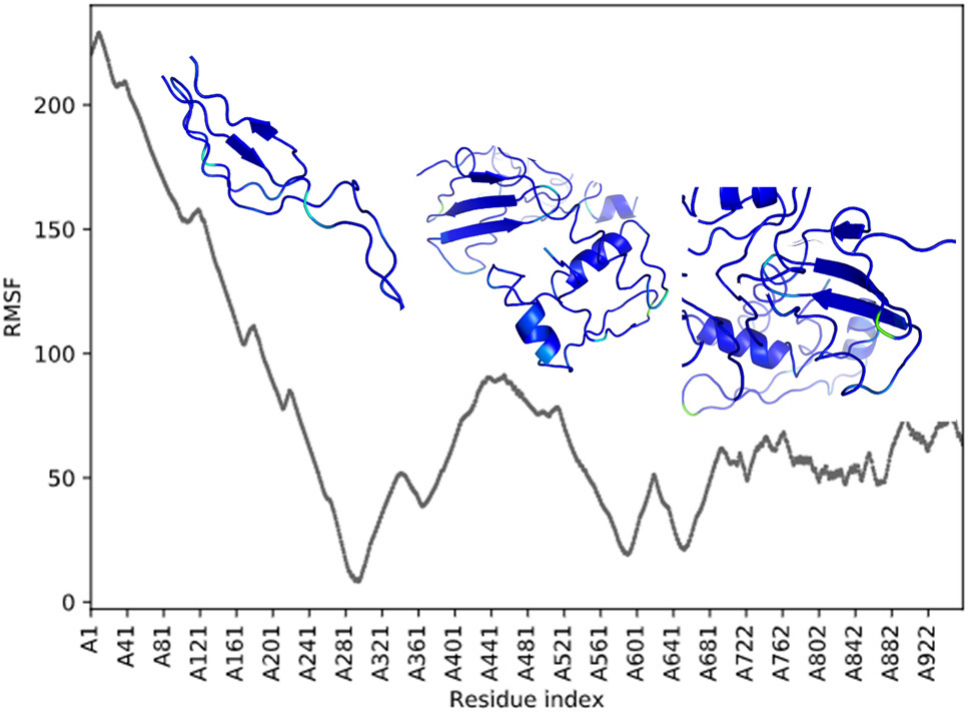
Root mean square fluctuation (RMSF) plot of the refined POTEE structure post-REMD simulation.

Root-mean-square deviation (RMSD) analysis showcases many residual disturbances that are present in the POTEE structure during the simulation that dictate its stability via confirming the equilibration [41]. A greater disturbance between trajectories was noted that therefore impacted the root-mean-square deviation (RMSD) of the replicas. At 10 ns, the RMSD values were recorded as main loops, helices, and beta strands were present in this region suggesting major changes in the refined POTEE structure when compared to the modeled one. The accuracy score describes the changes in the backbone of the original structure with the refined structure. Post molecular dynamic simulations, it is evident that the accuracy of the refined POTEE model is better when compared to modeled POTEE structure (refer to Table 6). The MolProbity score gives an idea about the atom–atom mapping in tertiary structures to look for clashes that may arise because of MD simulation problems within the structure and the dihedral angles. Usually, MolProbity scores lie in the range of 1–2 Å (A). Our results discern that refined POTEE (MolProbity score = 2.69) has been aligned better and has fewer clashes when compared to the originally submitted modeled POTEE (MolProbity score = 2.32). The radius of gyration (ROG) of a tertiary model defines the root-mean-square average of the distance of all atoms from the center of mass of the tertiary model [42]. The radius of gyration (ROG) is recorded to be less for the refined POTEE model (21.01 ± 1.00) when compared to the original modeled POTEE (21.86 ± 1.78) (refer Table 6). Figure 7 represents the RMSF plot with detailed regions of the residues that had higher fluctuation peaks and lower fluctuation peaks. The higher fluctuations were mainly observed in the highly coiled and super loopy regions starting from residues 1-246, while lower peaks were obtained in helices and beta-stranded residue regions.

**Table 6:** REMD and important electrostatics computations for POTEE structure.

Name	Accuracy	RMSD	MolProbity	Steric	Generalized Born	Coulomb	Electrostatic	Total	Radius
	score			hindrance score	self energy (GBSE) (kJ)	energy (kJ)	solvation energy (kJ/mol)	energy (kJ/mol)	of gyration (ROG)
*ModeledPOTEE*	0.9233	0.41 ± 0.22	2.32	31.0	−14,352.72 ± 152.95	−97,231.40	−2045.70	−97,773.65	21.86 ± 1.78
*Refined POTEE*	0.9587	0.39 ± 0.21	2.69	23.8	−14,599.90 ± 144.2	−97,887.60	−1979.55	−97,998.25	21.01 ± 1.00

**Figure 8:**
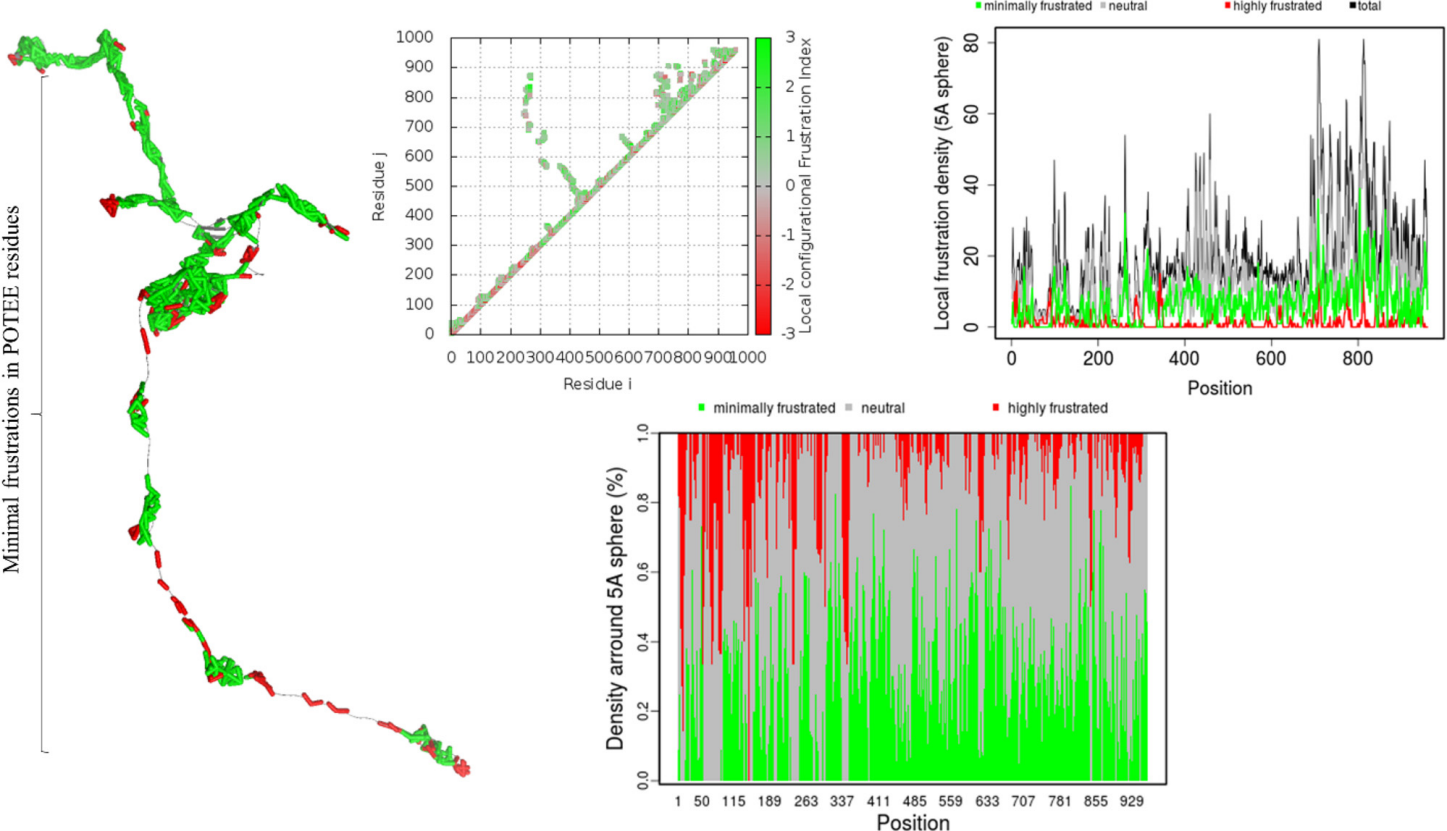
Macromolecular frustration in POTEE post REMD simulation.

After MD simulation, there is an energy landscape difference that dictates the refinement and further alterations in our modeled POTEE structure. The *macromolecular frustration* phenomenon is used to infer the functional dynamics and behavior of protein structures. The greater the frustrated regions, the greater the functional and binding cavities are present in a protein structure. Figure 7 encapsulates the combined, minimal, maximal, and neutral frustrations of the POTEE refined structure along with the density of frustration at various residues computed for 5 Å spheres. Maximal frustrations were present in residues 1-337 that mainly consist of loops while minimal frustrations were observed in 340-855 residues that form the helices and beta-strands in the POTEE tertiary model. The contact map visualization (refer Figure 8) also verifies the maximal and minimal frustrations in the initial residues and ending residues of the POTEE structure.

**Figure 9:**
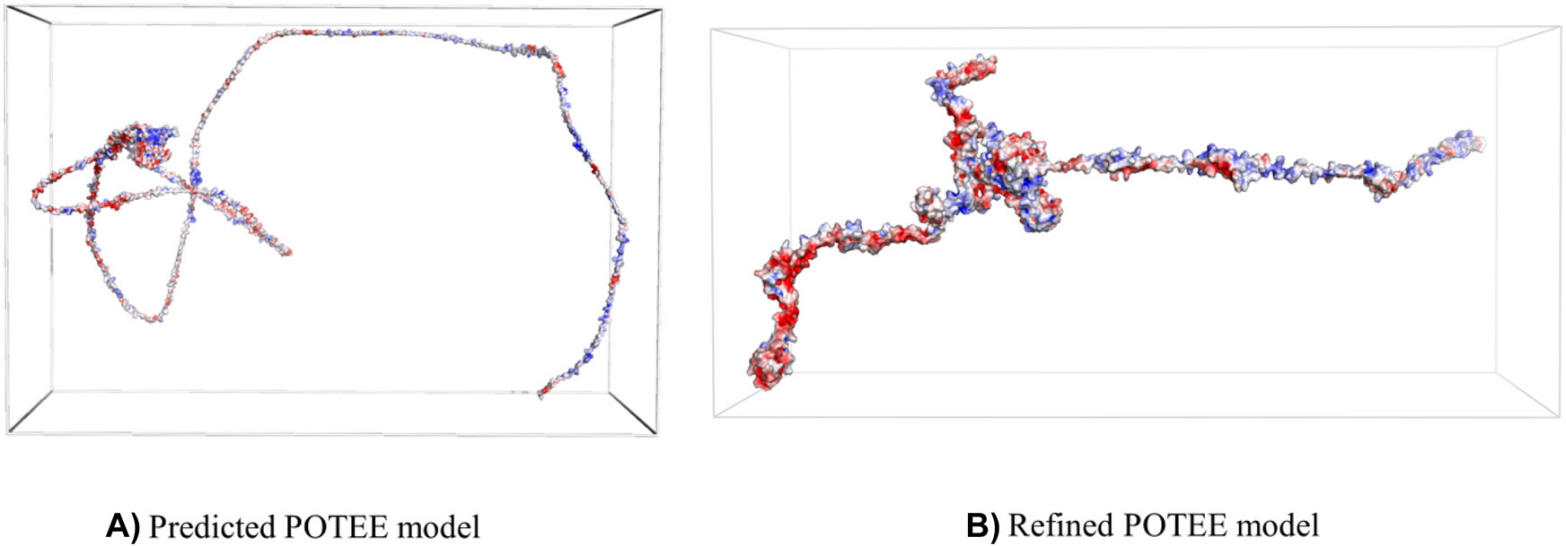
Electrostatics of POTEE represented in APBS maps. (A) Predicted POTEE model. (B) Refined POTEE model.

It is important to check how biomolecules associate with each other under various environments. That is where electrostatics plays a pivotal role in protein structural analyses. The adaptive Poisson–Boltzmann solver (APBS) provides solutions to the equations of continuum electrostatics for large biomolecules [43]. Our study reveals that refined POTEE structure had an APBS range in between −203.276 and 199.204, while the original modeled POTEE structure ranged in between −119.164 to 84.203 respectively. The molecular mechanics generalized Born surface area continuum solvation (MM-GBSA) indicate that post MD simulation, POTEE structure has become more stable, with fewer steric clashes, and is electrostatically stable. Figure 9 represents the MM-GBSA calculations in the form of an APBS mapin PyMOL [33] software for both the modeled and refined POTEE structures.

### 3.5 Network analysis

Recent studies have discerned that POTEE paralog gets epigenetically regulated in many cancers including ovarian cancer [19, 20, 44], [45], [46]. Sharma et al. (2019) [19] report that pericentromeric activation, global and locus-specific L1 hypomethylation, and loci-specific 5′ CpG hypomethylation when combined trigger the greater expression of POTE in high grade serous ovarian cancer (HGSOC) [19]. Shen et al. (2019) suggest that POTEE paralog promotes colorectal cancer by upregulating the SPHK1/p65 signaling pathway [45]. Another study reveals that POTEE, ApoA1, and HPX genes get upregulated in breast cancer and could be seen as a potential novel biomarker for the same [46], whereas, Wang et al. (2015) [20] discern that POTEE is hypomethylated in non-small cell lung cancer (NSCLC) and is associated with TNM NSCLC patients. All these recent studies suggest that POTEE paralog gets epigenetically activated in different cancers, however, there is no significant data available to prove its epigenetic association in terms of network-based epigenetic interactor analyses.

**Figure 10:**
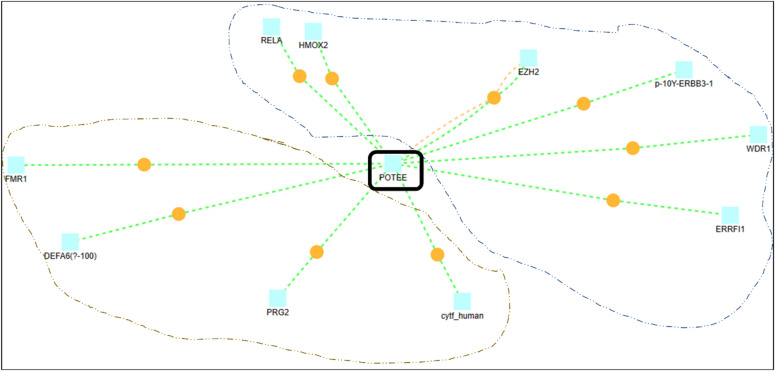
Network dynamics and interactors. Using the *k*-means clustering algorithm the 10 interactors have been classified into two groups demarcated in blue and orange respectively. Yellow pointer refers to direct and physical interactions of these associators with POTEE.

With different literature evidence, we know POTEE gets epigenetically regulated in cancers, but what we don’t know is why it gets epigenetically triggered. Therefore, it becomes necessary to analyze the POTEE sequence and to understand its significant associators and their behavior in different cancers. Therein, by deploying a network-based epigenetic analysis, we identified 200+ direct and indirect, inter-related, physical, and text-backed associators linked to POTEE. However, we selected only 10 highly significant, direct, and physical associators that had a confidence score of ≥5.0. These 10 associators were – RELA, HMOX2, EZH2, p-10Y-ERBB3-1, WDR1, ERRFI1, PRG2, FMR1, DEFA6-(?-100), cytf_human respectively. Figure 10 represents the network of these 10 associators and POTEE. To further make it lucid, we applied the *k*-means clustering algorithm to group closest and similar associators to the POTEE paralog. Two distinguishable groups were formed, Group A (demarcated in blue, see Figure 9) encapsulated – RELA, HMOX2, EZH2, p-10Y-ERBB3-1, WDR1, ERRFI1, whereas, group B (demarcated in orange) consisted of PRG2, FMR1, DEFA6-(?-100), cytf_human. Table 7 provides a brief description of the 10 identified interactors.

**Table 7:** 10 highly significant, direct and physical network associators of POTEE.

Name	Description
RELA	Proto-oncogeneNF-KB subunit
HMOX2	Heme oxygenase-2
EZH2	Enhancer of Zeste 2 polycomb repressive complex 2 subunit
p-10Y-ERBB1	Epidermal growth factor receptor
WDR1	WD repeat containing protein-1
ERRFI1	ERBB receptor feedback inhibitor-1
Cytf_human	Cystatin F (human)
PRG2	Proteoglycan-2
FMR1	FMRP translational regulator-1
DEFA6 (?-100)	–

On manual text mining the literature evidence, we found that out of 10, 8 of these associators were epigenetically modified and regulated in different diseases. Group A associators overpowered the epigenetic link to group B interactors. RELA has shown an increased methylation level that is significant in the progression of breast cancer [47], HMOX2 has shown an increased hypomethylation in endometriosis [48], while EZH2 mediates histone modification H3K27m3 and causes several cancers [49]. ERRFI1 is discerned to have an epigenetic downregulation in neuroblastoma tumors [50], and WDR1 has been shown to get overexpressed in non-small cell lung cancer (NSCLC) [51], whereas, p10Y-ERBB3-1 is discerned to have shown histone methylation of H3K27m3 in general [52]. From group B, we could identify only FMR1 and PRG2 that show epigenetic regulations. FMR1 is shown to have regulated histone methylation H4K27m3 in lymphoblastoid and fibroblast cell lines [53] while PRG2 has been discerned to get hypomethylated in acute myeloid leukemia [54]. This evidence suggests that since the majority of the network associators of POTEE are epigenetically activated in many cancers, it is quite natural for POTEE paralog to get over-expressed and epigenetically regulated in ovarian cancer too. Moreover, there exist various experimental studies [19, 20, 44], [45], [46], [47], [48], [49], [50], [51], [52], [53], [54] that discern its epigenetic dynamics in different diseases.

## 4 Discussion

The cancer testis antigen (CTA) family member – prostate ovary testis embryo expression (POTE) is a class of genes that have been discerned to play a pivotal role in many diseases especially cancers. Because of limited literature, there is no experimental or derived structure of POTEE paralog. Also, the lack of genomic information makes it crucial to deduce pivotal information that can be used as a lead to identify and understand its epigenetic trigger that leads to ovarian cancer in females. With the aid of an exploratory modus operandi, we identified six main matching motifs that are present in the mRNA sequence of POTEE paralog, out of them, three motifs – (i) CTTCCAGCAGATGTGGATCA (score 13.1291), (ii) GGAACTGCC (score 12.1573), (iii) CGCCACATGCAGGC (score 8.44245) are most probable candidates to be in the nucleotide sequence of POTEE as these were matching to other motifs already known to be ubiquitous in established and validated repositories. Also, A–T pair was 0.2534 and nucleotide pair C–G was 0.2466 in %age as computed in the POTEE mRNA sequence. These motifs were perfect matches to various present in the human and mouse genome. Moreover, these motifs were matches to zinc finger factors, DNA-binding domains, and transcription factors (TFs) present in both the human and mouse genome. There were 64,488 motif occurrences with a *p*-value less than 0.0001. The best match motif was identified to be CTTCCAGCAGATGT.

In order to predict the tertiary structure, instead of adopting the traditional approach, we deployed the template-based modeling (TBM) method that utilized the power of deep neural network (DNN) learning. There is a significant difference between the Swiss-Model POTEE structure and our DNN-based POTEE model. The predicted model has been developed using the best matching PDB template 6I4D_A. The predicted model of POTEE has a single chain A with a length of 1-960 residues and encapsulates the major domains and motifs of the POTEE paralog. Also, the main regions of functionality start from residue 248 and end at residue 960. Loops are formed from residue 1-246. After structure alignment, it is evident that our predicted model of POTEE and the Swiss-Model actin POTEE region that starts from 705-1075 were aligned mainly at 5 intersections; residues 6-12 of predicted POTEE was perfectly aligned to 706-712. Resides 26-28, 66-76, and 81-140 in the predicted POTEE model were perfectly aligned to 726-728, 767-777, and 781-841 residues of the Swiss-Model POTEE structure. The longest aligned match portion started from residue 179-250 in our predicted model of POTEE with residue 879-949 of the Swiss-Model POTEE structure. To validate our predicted model, we utilised AlphaFold [40] structure predicted POTEE model and thus aligned the two structures to check how well our predicted model has been developed. It was found that there was a perfect alignment to both the structures from residue 237-958, that corresponds to the fact that the stretch of our predicted model has a very high confidence per residue i.e., >90. While, residues starting from 1-236 and stretch of residue starting from 959-1071 didn’t align well, that simply refers to having a poor per residue confidence score, i.e., <50.

Post-REMD, the POTEE model has been refined with its overall energy being stable along with a good overall root mean square deviation (RMSD) score with less steric clashes. Root-mean-square deviation (RMSD) analysis showcases many disturbances that are present in the POTEE structure during simulation dictating the stability by confirming the equilibration. A greater disturbance between trajectories was noted in the RMSD of the replicas. The accuracy of the refined POTEE model is better when compared to modeled POTEE structure (refer to Table 6, the second column). Our results discern that refined POTEE (MolProbity score = 2.69) has been aligned better and has fewer clashes when compared to the originally submitted modeled POTEE (MolProbity score = 2.32). The higher fluctuations were mainly observed in the highly coiled and super loopy regions starting from residues 1-246, while lower peaks were obtained in helices and beta-stranded residue regions. The molecular mechanics generalized Born surface area continuum solvation (MM-GBSA) indicate that post MD simulation, POTEE structure has become more stable, with fewer steric clashes, and is electrostatically stable.

The network-based epigenetic analysis discerns only 10 highly significant, direct, and physical associators that had a confidence score of ≥5.0 and were namely– RELA, HMOX2, EZH2, p-10Y-ERBB3-1, WDR1, ERRFI1, PRG2, FMR1, DEFA6-(?-100), cytf_human respectively. Since the majority of the network associators of POTEE are epigenetically activated in many cancers as they have been reported in the literature, it is quite natural for POTEE paralog to get over-expressed and epigenetically regulated in ovarian cancer too. Additionally, we conclude that although there are a few studies that have shown POTEE gets hypomethylated (over-expressed) in ovarian cancer, but our study has validated this theory using an *in-silico* analysis that uses genomic, structural, electrostatic and epigenetics-based network approach.

## 5 Conclusions

With an exhaustive and an exploratory analysis, we would like to conclude that POTEE paralog has motifs that are matches to zinc finger factors, DNA-binding domains, and transcription factors (TFs) ubiquitousin both the human and mouse genome. The best match motif was identified to be CTTCCAGCAGATGT. There are a few studies that have shown a correlation between transcription factor BORIS (Brother of Regulator of Imprinted Sites) viz., paralogous to the well characterized, highly conserved, multivalent 11 Zn-finger factor CTCF but are different and –N and C termini. BORIS and POTE both come from a cancer testis antigen (CTA) family, and there are a few studies that showcase BORIS directly dictates CTA gene expression regulation [55–58]. Additionally, the BORIS/CTCF mRNA expression ratio is also linked with DNA hypomethylation in cancers. Our genomic analysis thus points out the direct correlation of the CTCF motif identified in POTEE mRNA sequence could be a useful lead in understanding why it gets hypomethylated in ovarian cancer. The predicted model has been developed using a deep-learning based homology modelling approach with the best matching PDB template 6I4D_A. and has a single chain A with a length of 1-960 residues encapsulating domains, motifs, and loops. Also, the main regions of functionality start from residue 248 and ends at residue 960. Loops are formed from residue 1-246. To validate our predicted POTEE model, we used AlphaFold POTEE structure. It was observed that there was a perfect alignment to both the structures (predicted POTEE & AlphaFold POTEE model) from residue 237-958 referring to a high confidence per reside (>90) of our predicted model. Post molecular dynamic simulations and related analyses such as – molecular mechanics generalized Born surface area continuum solvation (MM-GBSA) indicate that POTEE structure has become more stable, with fewer steric clashes and is electrostatically stable and that the higher fluctuations were mainly observed in the highly coiled and super loopy regions starting from residues 1-246, while lower peaks were obtained in helices and beta stranded residue regions. There are 10 highly significant, direct and physical associators with a confidence score of ≥5.0 namely – RELA, HMOX2, EZH2, p-10Y-ERBB3-1, WDR1, ERRFI1, PRG2, FMR1, DEFA6-(?-100), cytf_human and the majority of the network associators of POTEE are epigenetically activated in many cancers. Thus, it is quite natural for POTEE paralog too to get over-expressed and epigenetically regulated in cancer and to be specific, ovarian cancer. Thus, it can be seen as a positive prognostic indicator to diagnose ovarian cancer in its early stages.

## Abbreviations used:

APBS: adaptive Poisson–Boltzmann solver
BORIS: brother of regulator of imprinted sites
CTCF: CCCTC-binding factor
CTA: cancer testis antigen
DNMT: DNA methyltransferases
DNN: deep neural network
FIMO: find individual motif occurrence
HDAC: histone deacetylases
HMT: histone methyltransferases
MMGBSA: molecular mechanics/generalized Born surface area
NSCLC: non-small cell lung cancer
POTE: prostate ovary testis embryo expression
POTEE: prostate ovary testis embryo expression paralog E
REMD: replica exchange molecular dynamic simulation
RMSD: root mean square deviation
RMSF: root mean square fluctuation
ROG: radius of gyration
TBM: template based modelling
WHO: World Health Organization
