# Supplementary material for: In silico approach to understand epigenetics of POTEE in ovarian cancer
Source: J Integr Bioinform. 2021 Nov 18;18(4):20210028. doi: 10.1515/jib-2021-0028 (PMC8709732; doi:10.1515/jib-2021-0028)
Supplement: Supplementary file 1 [file jib-18-20210028-s001.docx]

**Table S1. MOTIF ENRICHMENT FOR M1, M2 AND M5 PRESENT IN HUMAN mRNA POTEE SEQUENCE.**

| **Query Motif** | **Motif Name** | **e- value** | **p-value** | **q-value** | **Orientation** | **Overlap** | **Match Logo** |
| --- | --- | --- | --- | --- | --- | --- | --- |
| **M1** | MA0139.1 (CTCF) | 3.14e-01 | 1.74e-04 | 5.69e-01 | Reverse | 14 | **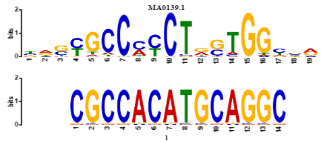** |
|  | MA1100.1 (ASCL1) | 1.59e+00 | 8.82e-04 | 5.69e-01 | Reverse | 13 | **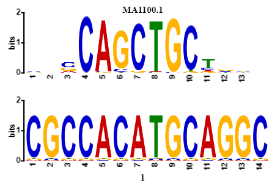** |
|  | MA0750.2 (ZBTB7A) | 1.67e+00 | 9.23e-04 | 5.69e-01 | Reverse | 13 | **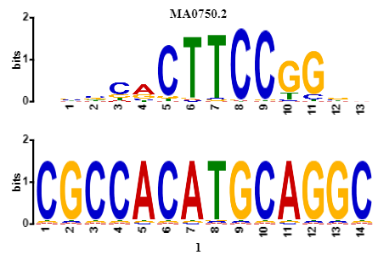** |
|  | HES5_DBD_2 | 2.32e+00 | 1.28e-03 | 5.69e-01 | Reverse | 12 | **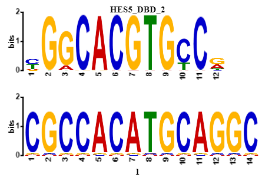** |
|  | MA0147.3 (MYC) | 2.53e+00 | 1.40e-03 | 5.69e-01 | Normal | 12 | **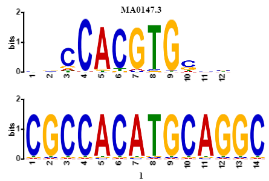** |
|  | MA0003.3 (TFAP2A) | 2.60e+00 | 1.44e-03 | 5.69e-01 | Normal | 11 | **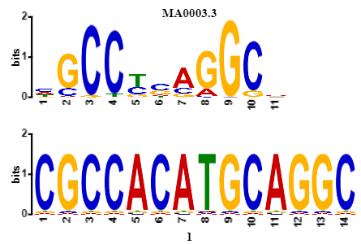** |
|  | TFAP2A_DBD_2 | 2.60e+00 | 1.44e-03 | 5.69e-01 | Normal | 11 | **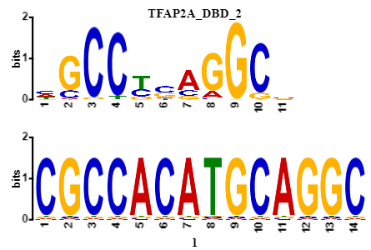** |
|  | MA0095.2 (YY1) | 9.04e+00 | 5.00e-03 | 6.18e-01 | Normal | 12 | **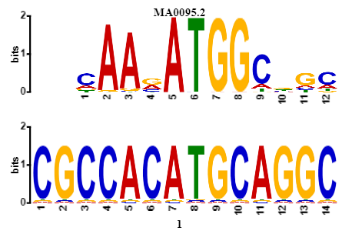** |
| **M2** | MA0062.2 (Gabpa) | 1.16e+00 | 6.40e-04 | 1.00e+00 | Normal | 9 | **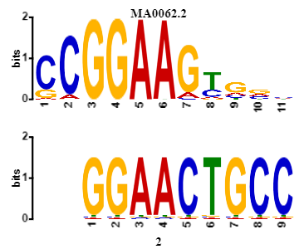** |
|  | MA0750.2 (ZBTB7A) | 2.12e+00 | 1.18e-03 | 1.00e+00 | Normal | 9 | **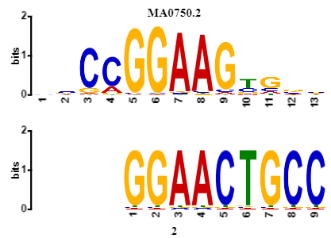** |
|  | MA0107.1 (RELA) | 3.47e+00 | 1.92e-03 | 1.00e+00 | Reverse | 9 | **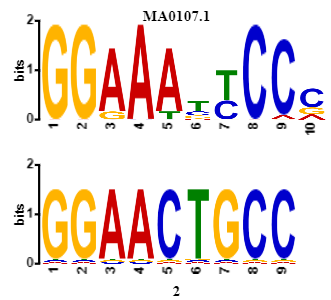** |
|  | MA0100.3 (MYB) | 3.67e+00 | 2.03e-03 | 1.00e+00 | Normal | 9 | **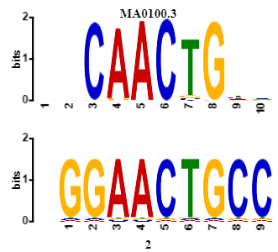** |
| **M5** | ELK1_full_2 | 7.71e-01 | 4.26e-04 | 9.45e-01 | Reverse | 15 | **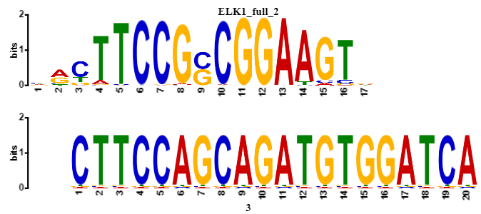** |
|  | UP00046_1 (Tcfe2a_primary) | 1.77e+00 | 9.77e-04 | 1.00e+00 | Reverse | 17 | **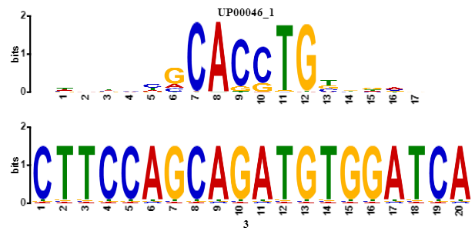** |
|  | MA1100.1 (ASCL1) | 4.14e+00 | 2.29e-03 | 1.00e+00 | Normal | 13 | **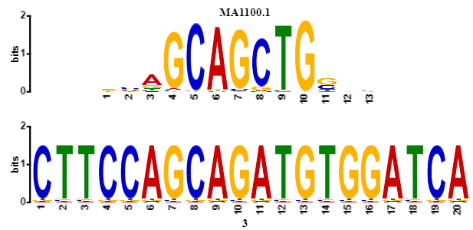** |
|  | MA0830.1 (TCF4) | 5.58e+00 | 3.08e-03 | 1.00e+00 | Reverse | 10 | **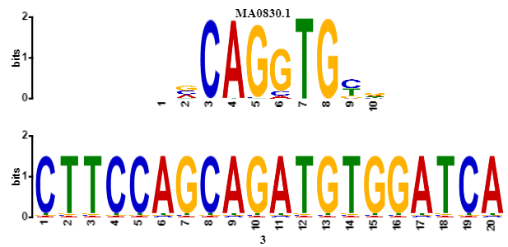** |
|  | MA0130.1 (ZNF354C) | 5.69e+00 | 3.15e-03 | 1.00e+00 | Reverse | 6 | **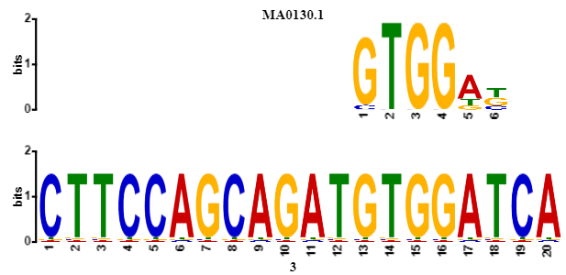** |
